# Individual- and community-level correlates of intermittent preventive treatment of malaria in pregnancy in Ghana: further analysis of the 2019 Malaria Indicator Survey

**DOI:** 10.1007/s43999-024-00058-6

**Published:** 2024-12-23

**Authors:** Jacob Owusu Sarfo, Patience Fakornam Doe, Dickson Okoree Mireku

**Affiliations:** 1 Department of Health, Physical Education and Recreation, University of Cape Coast, Cape Coast, Ghana; 2 Centre for Behaviour and Wellness Advocacy, Koforidua, Ghana; 3 School of Nursing and Midwifery, University of Cape Coast, Cape Coast, Ghana; 4 Directorate of Academic Planning and Quality Assurance, University of Cape Coast, Cape Coast, Ghana

**Keywords:** Intermittent preventive treatment of malaria in pregnancy, Antennal visit, Malaria, Malaria indicator survey, Pregnant women, Ghana

## Abstract

**Background:**

Ghana adopted the policy on Intermittent Preventive Treatment of malaria in pregnancy using Sulfadoxine-Pyrimethamine (IPTp-SP) in 2004. Notwithstanding the government’s and other stakeholders’ efforts in Ghana, optimal uptake (three or more doses of IPTp-SP) has slightly declined since 2016. The study examined the individual and community-level correlates of pregnant women who take optimal or none/partial doses (less than three doses) of IPTp-SP using the Ghana Malaria Indicator Survey (GMIS) 2019.

**Methods:**

We conducted a secondary analysis of the GMIS 2019 data. Our analytical sample included 1,151 women aged 15-49 with their most recent birth in the last two years before the survey.

**Results:**

The overall uptake among participants was approximately 8.2% for none, 30.15% for 1–2 (partial), and 61.6% for 3 or more (optimal) doses of IPTp-SP. The level of uptake differs depending on the individual rather than community-level characteristics of pregnant women. Individual-level demographic factors— residents in Upper East (OR 3.0, 95% CI; 1.2–7.3) and Upper West (OR 5.3, 95% CI; 1.9–14.7) —and health-related factors—the four or more antenatal (ANC) visits (OR 3.3, 95% CI; 1.8–6.0) were associated with optimal IPTp-SP uptake among pregnant women in Ghana. However, late scheduling of the first ANC visit in the second trimester (OR 0.7, 95% CI; 0.5–1.0)— predicted less IPTp-SP uptake.

**Conclusions:**

Few regions (Upper East and West) are doing better than the capital, Greater Accra Region, in terms of optimal uptake. Also, early scheduling of ANC in the first trimester and increased ANC attendance are key for increased uptake. There is a need for policy, interventions, and research on malaria prevention in pregnancy to improve the decline in uptake.

## Introduction

### Background

Malaria and related adverse outcomes in pregnancy are a significant threat to global maternal health. Out of the total global population, over 95% of malaria cases can be found in the World Health Organization (WHO) African Region [1]. Among the WHO African region, four countries in sub-Saharan Africa (SSA) alone account for 48% of the global malaria cases [1]. Among the worst affected by malaria in SSA are children and pregnant women [2–6]. In an attempt to mitigate the prevalence of malaria in SSA, several countries, including Ghana, have implemented several interventions and policies to reduce malaria prevalence among pregnant women. The impacts of these interventions and policies have shown a steady decline by 2019 [7]. 

In the case of Ghana, several studies from the past decade have shown that malaria and its related adverse outcomes, such as anemia, have severe consequences for human health and socioeconomic development [8]. In collaboration with international funding organizations, Ghana’s government has prioritized efforts focusing on host susceptibility, parasite, and malaria vector (mosquitoes) control interventions like insecticide-treated bed nets (ITN), social and behavior change communication (SBCC) messages, effective case management, chemoprophylaxis, insect repellents, and dredging up standing water [9, 10]. Among these interventions, the use of ITN, SBCC messages and chemoprophylaxis for preventing malaria have been emphasized in Ghana as part of the National Malaria Control Program for pregnant women [10–13]. The ITN use in Ghanaian households following the free mass distribution initiative in 2010 showed that pregnant women increased usage from 32.6% to 49.7% in 2011 and 2017, respectively [12]. In addition, exposure to SBCC messages on malaria prevention and early management among pregnant women has shown an increasing trend over the years [13]. Also, the level of pregnant women’s exposure to SBCC messages in Ghana has been shown to correlate significantly to ITN usage [14] and chemoprophylaxis for preventing malaria uptake [10]. 

In the 1990s, the main chemoprophylaxis for preventing malaria during pregnancy among pregnant women in sub-Saharan countries was the weekly administration of chloroquine prophylaxis throughout Antenatal Care (ANC) [11, 15]. Given the poor adherence to chloroquine and the parasite’s tolerance to the drug in SSA, this intervention was proven unsuccessful in pregnant women [15, 16]. Consequently, the WHO introduced daily or weekly doses of intermittent preventive treatment with sulfadoxine-pyrimethamine (IPTp-SP) in 2000 to replace the existing chloroquine chemoprophylaxis program for pregnant women [17–20]. According to WHO [19], three or more doses of IPTp-SP are recommended for every pregnant woman in malaria-endemic regions. All information that was obtained, including data, was kept confidential. Following this recommendation, Ghana adopted the IPTp-SP use for pregnant women policy in 2004, later updated in 2007, 2012, and 2017 [20]. 

For example, the initial 2004 policy recommended three doses of IPTp-SP from 16 weeks of pregnancy every month through a Directly Observed Therapy administration. By 2012, at least two doses of IPTp-SP were provided to pregnant women at least once a month, with at least a month interval. After 2012, other updates by the National Malaria Control Programme, Ghana integrated IPTp-SP as part of national antenatal care and improved its uptake in 2014 [20]. This update, reinforced in 2016, was in response to ongoing evaluations and WHO that required a minimum of three doses and allowed for up to five doses during pregnancy. This regimen still guides IPTp-SP use during pregnancy.

Despite IPTp-SP use during pregnancy being clinically successful, adherence among pregnant women in Ghana has not reached its optimum use of more than three doses [10, 19, 21]. According to Darteh et al. [10], expectant mothers were influenced by socio-demographic factors like their age, region of residence, ethnic background, and religion. For example, unlike the other regions, pregnant women from the Northern Region had a higher likelihood of taking optimal uptake of IPTp-SP [10]. Also, Pentecostals/charismatics had a higher relative risk of taking partial doses compared with Protestants [10]. Furthermore, inadequate supply of medication, ineffective behaviour change messages on malaria treatment and ANC staff’s knowledge of the protocol also affected adherence to IPTp-SP among pregnant women in Ghana [10, 21]. 

Aside from the individual-level factors of pregnant women, community characteristics of these women in some African studies have significantly influenced ANC attendance and IPTp-SP uptake. For example, studies conducted in Burkina Faso [22] and Uganda [23] have shown that using Community Health Workers to identify pregnant women in the community and encouraging them to attend ANC and community IPTp-SP distribution tend to facilitate early initiation of ANC and increase IPTp-SP coverage. Also, female residents in urban areas were less likely to have at least three doses because they perceived themselves as less susceptible to malaria during pregnancy. Also, less-resourced communities and regions were less likely to have three or more doses of IPTp-SP [22, 23]. A quasi-experimental study to evaluate the impact of community delivery of intermittent preventive treatment of malaria in pregnancy on its coverage in four SSA countries, including the Democratic Republic of the Congo, Madagascar, Mozambique, and Nigeria, supported previous findings that community distribution of IPTp-SP was associated with higher uptake without a decrease in ANC attendance [24]. Although these studies suggested the importance of community-context factors in the Sub-Saharan Region, little is known about these correlates in Ghana.

As Ghana works to attain the Sustainable Development Goal (SDG Goal 3) to reduce maternal mortality by 2030, 80% of pregnant women received partial doses (at least two doses) of IPTp-SP [25]. Our extensive literature review found little recent evidence exploring maternal socio-demographic and health-related characteristics associated with IPTp-SP uptake among pregnant women in Ghana using a national representative sample. Most of these existing studies have offered essential demographic risk correlates and other characteristics like knowledge-related factors, ANC attendance, and gestational age at ANC visits [11, 26, 27]. Nevertheless, Chikwasha et al. [28] noted that the uptake of IPTp-SP in Zimbabwe was affected by pregnant women’s socio-demographic and health-related factors. Thus, there is a gap in examining, in addition to the individual factors, key community characteristics that may be associated with IPTp-SP uptake among pregnant women in Ghana are effectively embraced. Additionally, WHO [19] pointed out that IPTp-SP acceptance has been shown to reduce the rate of infant mortality and pregnancy-related complications globally. As Ghana seeks to attain Sustainable Development Goal 3 by 2030, the uptake of IPTp-SP will help the nation to end malaria and child mortality and reduce maternal deaths due to pregnancy-related complications. Our paper examined the individual and community-level factors of pregnant women who take optimal or none/partial doses of IPTp-SP using Ghana’s 2019 Malaria Indicator Survey.

### Research question

What individual- and community-level factors in Ghana predict the uptake of 3 or more doses of IPTp-SP among pregnant women?

### Conceptual framework

**Fig. 1:**
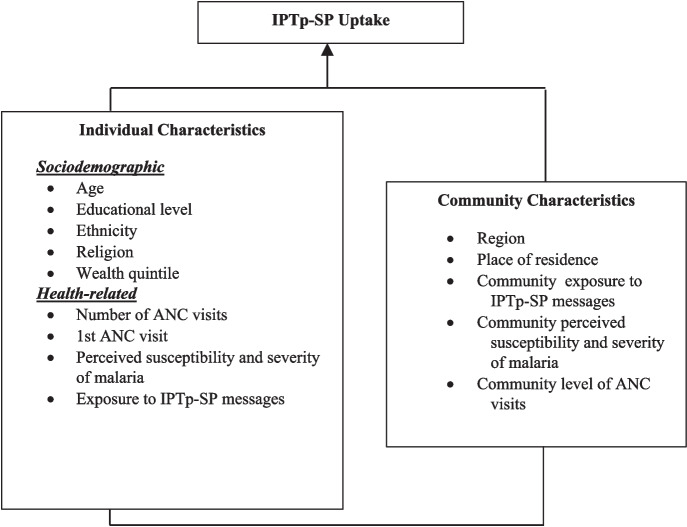
Conceptual framework

Figure 1 shows the dependent and independent variables in the study’s conceptual framework. The dependent variable in this study involves the pregnant women’s IPTp-SP uptake in Ghana, categorized in this research as none/partial and optimal doses. The none/partial dose category consists of those who take less than three or no doses of IPTp-SP, while an optimal dose is at least three or more doses [10, 19].

Also, our study has two independent variables: individual- and -community-level factors. The individual-level characteristics of pregnant women comprised socio-demographic factors (age, education, ethnicity, religion, region of residence, and wealth quintile, which were calculated based on household assets, characteristics, and living conditions) and health-related factors (health insurance coverage, number of ANC visits, 1st ANC visit, poorly perceived susceptibility and severity of malaria, and exposure to IPTp-SP messages) [10, 28, 29]. 

Based on previous literature, we also conceptualized that the community-level characteristics of pregnant women, such as place of residence, community exposure to Malaria messages, perceptions of community susceptibility and severity of malaria, and community level of ANC visits, could influence IPTp-SP uptake of pregnant women in Ghana [11, 28–30]. 

## Methods

### Data

We used data from the Ghana Malaria Indicator Survey 2019 (GMIS 2019). The survey used Ghana’s 2010 Population and Housing Census sampling frame in the ten existing administrative regions. The GMIS 2019 sample was created through two rounds of stratification and selection from the sampling frame. After dividing respective regions into 200 enumeration areas (97 urban and 103 rural areas), 20 strata were formed for sampling. In each stratum, separate census enumeration areas sample selections were made in two phases [25]. Following a stratified two-stage cluster design, interviews were open to all women between the ages of 15 and 49 who were either long-term residents or guests the night before the survey of the selected households.

With a 99% response rate, a total of 5,181 eligible women were interviewed at the end of the survey [25]. In this current study, our analytical sample included 1,151 women aged 15–49 with their most recent birth in the last two years before the survey. Also, these women had received varied doses of IPTp-SP, ranging from none to nine doses. The GMIS 2019 protocol was approved by both the ICF Institution Assessment Committee and the Ghana Health Ethics Review Board. The public can get the dataset by visiting https://www.dhsprogram.com/data/dataset/Ghana_MIS_2019.cfm?flag=0.

### Variables

#### Dependent variables

We selected women’s use of IPTp-SP during pregnancy as the outcome variable for the study. We constructed this variable from two survey items: “During this pregnancy, did you take SP/Fansidar to prevent you from getting malaria?” and “How many times have you received SP/Fansidar during this pregnancy?” Women’s responses regarding the doses of SP/Fansidar received during pregnancy were dichotomized into none/partial (< 3 times) = 0 and optimal (3 + times) = 1.

#### Independent variables

The explanatory variables comprised two primary contextual categories: maternal individual and community characteristics. The individual-level characteristics of pregnant women included socio-demographic and health-related factors. Under the socio-demographic factors, the age of women was categorized within a 5-year interval (15–19, 20–24, 25–29, 30–34, 35–39, 40+). Educational level was also classified into none, primary, and secondary and higher levels. Ethnicity was in four categories: Akan, Mole-Dagbani, Ewe, and others. The religion of participants was categorized into catholic, pentecostal/charismatic, other Christian, Muslim, and others. Additionally, participants’ wealth quintile was classified into five (poorest, poorer, middle, richer, and richest). Under the health-related factors, we also selected and categorized the following variables: number of ANC visits at pregnancy (0–3, 4–7, and 8+), scheduling of 1st ANC visit (0–3, 4–6, and 6–9 months). The perceived susceptibility and severity of malaria were constructed from participants’ belief that they don’t worry about malaria because it can be easily treated (agree = 0 and disagree = 1). We also created the variable “exposure to IPTp-SP messages” from the responses to the item that measured whether pregnant women have seen or heard messages in the past 6 months that they should take drugs to prevent malaria (yes = 1 and no = 0). Additional community-level explanatory variables encompassed the participants’ region of residence based on the original ten regions of Ghana, place of residence (rural and urban), community exposure to malaria messages, community perception of susceptibility and severity of malaria, and community level of ANC visits. Using a STATA command, we created these community-level variables by constructing aggregate values based on the clusters in the survey based on a statistical code provided by the Demographic and Health Survey team.

### Statistical analysis

We conducted all our statistical analyses using STATA version 17. All items were categorical and assigned respective codes before final descriptive and inferential statistics were conducted except for these three community-level factors (community exposure to malaria messages, Poorly Perceived Community Susceptibility and Severity of Malaria, and community level of ANC visits). We used descriptive and inferential statistics for our data analysis. Explicitly, the Chi-Square (χ^2^) statistic was used to examine the associations between individual and community-level characteristics and IPTp-SP uptake. We used binary logistic regression to identify the factors that predict IPTp-SP adoption at both none/partial and optimal levels. Before conducting actual statistical analysis, frequency distributions were weighted to correct the non-proportional distribution of the sample across different areas and the possibility of variances in response rates. Furthermore, we employed the survey command in STATA to account for the complex sampling design of the data in the binary logistic regression. Results from this analysis were shown as adjusted odds ratios (OR) with 95% confidence intervals (CIs) at *p* < 0.05.

## Results

### Demographic characteristics of participants

We used a total sample of 1,151 women between the ages of 15 and 49 with their most recent birth in the last two years before the survey for this data analysis. As shown in Table 1, about 71.8% of women were between the reproductive ages of 20 and 35 years old, with about 59.0% having Secondary and Higher Education. Approximately 40.9% of the women were Akans, with 76.6% being Christians. Greater Accra Region, the Capital of Ghana and the Ashanti Region had 31.0% responses due to the dense populations. Most women (55.9%) reported a wealth index between the middle and the richest. About 89.8% of women had previously visited the ANC clinic more than four or more times, while more than half (65.9%) went for their 1st antenatal check (months). Also, almost a balanced proportion of participants were selected from urban and rural settlements in Ghana.

**Table 1:** Demographic characteristics of participants

Variables	Number of participants (*n*)	Percent (%)
***Demographic Characteristics***		
**Age (in years)**		
15–19	88	7.7
20–24	245	21.3
25–29	334	29
30–35	244	21.2
35–39	173	15
40+	67	5.8
**Educational Level**		
None	224	19.5
Primary	248	21.5
Secondary and Higher	679	59
**Ethnicity**		
Akan	471	40.9
Mole-Dagbani	254	22.1
Ewe	171	14.9
Other	256	22.2
**Religion**		
Catholic	78	6.8
Pentecostal/Charismatic	509	44.2
Other Christians	294	25.6
Muslim	231	20.1
Other	39	3.4
**Region**		
Greater Accra	174	15.1
Central	98	8.5
Western	114	9.9
Volta	121	10.5
Eastern	111	9.7
Ashanti	184	15.9
Brong Ahafo	95	8.2
Northern	161	14
Upper East	55	4.8
Upper West	37	3.2
**Wealth Index**		
Poorest	242	21
Poorer	265	23.1
Middle	232	20.2
Richer	222	19.3
Richest	189	16.5
**Number of Antenatal Visits During Pregnancy**		
0–3 visits	117	10.1
4–7 visits	546	47.4
8 + visits	488	42.4
**Scheduling of 1st Antenatal Check (in months)**		
0–3 months	758	65.9
4–6 months	375	32.6
7–9 months	17	1.5
**Poorly Perceived Susceptibility and Severity of Malaria**		
Disagree	452	39.3
Agree	699	60.7
**Exposure to messages on IPTp-SP (in the past 6 months)**		
No	1,082	94
Yes	69	6
**Place of Residence**		
Urban	500	43.4
Rural	651	56.6
**Total**	1,151	100

Data presented included women aged 15–49 who had children under age 24 months and had sought ANC during their pregnancy by selected demographic characteristics, Ghana 2019 Malaria Indicator Survey

Source: 2019 GMIS dataset

### Bivariate analysis of pregnant women’s IPTp-SP uptake in Ghana

The bivariate analysis presented in Table 2 showed the results of the percentage of women between the ages of 15 and 49 with their most recent birth in the last two years before the survey, by their characteristics, who had received none/partial or optimal doses of IPTp-SP. IPTp-SP uptake was approximately 8.2% for none, 30.2% for 1–2 (partial), and 61.6% for 3 or more (optimal) doses. Due to the inadequacy of the no-dose group as a comparable case group of the optimal dose group, we categorized 2 groups instead: none/partial and optimal dose groups. From our analysis, the overall uptake among participants was 38.4% for none/partial doses (less than three doses of IPTp-SP) and 61.6% for optimal doses of IPTp-SP (three or more doses of IPTp-SP).

**Table 2:** Bivariate analysis of pregnant women’s IPTp-SP uptake

	< 3 Doses		3+ Doses		
Variables	%	CI	%	CI	*P value*
***Individual-Level: Socio-demographic***					
** Age (in years)**					0.148
15-19	48	[35.7-60.6]	52	[39.4-64.3]	
20-24	45.8	[38.8-53.0]	54.2	[47.0-61.2]	
25-29	36.2	[29.0-44.0]	63.8	[56.0-71.0]	
30-35	36.8	[28.9-45.5]	63.2	[54.5-71.1]	
35-39	37.2	[28.5-46.9]	62.8	[53.1-71.5]	
40+	28.2	[17.8-41.7]	71.8	[58.3-82.2]	
**Educational Level**					0.059
None	35.7	[26.6-46.0]	64.3	[54.0-73.4]	
Primary	48	[39.7-56.4]	52	[43.6-60.3]	
Secondary And Higher	36.7	[32.7-41.0]	63.3	[59.0-67.3]	
**Ethnicity**					0.127
Akan	36.8	[32.3-41.6]	63.2	[58.4-67.7]	
Mole-dagbani	34.6	[28.5-41.2]	65.4	[58.8-71.5]	
Ewe	41.4	[32.2-51.2]	58.6	[48.8-67.8]	
Other	45.7	[37.1-54.6]	54.3	[45.4-62.9]	
**Religion**					0.008
Catholic	25.6	[17.3-36.2]	74.4	[63.8-82.7]	
Pentecostal/Charismatic	45.1	[39.5-50.9]	54.9	[49.1-60.5]	
Other Christians	35.8	[30.1-42.0]	64.2	[58.0-69.9]	
Muslim	33.4	[26.7-40.8]	66.6	[59.2-73.3]	
Other	41.6	[23.8-61.8]	58.4	[38.2-76.2]	
** Wealth Index **					
Poorest	40.4	[31.7-49.7]	59.6	[50.3-68.3]	0.961
Poorer	38.5	[31.4-46.1]	61.5	[53.9-68.6]	
Middle	39	[32.5-45.9]	61	[54.1-67.5]	
Richer	36.7	[29.7-44.2]	63.3	[55.8-70.3]	
Richest	40.5	[31.5-50.1]	59.5	[49.9-68.5]	
***Individual-Level: Health-Related***					
**Number of Antenatal Visits During Pregnancy**					0.001
0-3 visits	71.9	[59.2-81.9]	28.1	[18.1-40.8]	
4-7 visits	41.4	[36.6-46.5]	58.6	[53.5-63.4]	
8+ visits	28.3	[23.8-33.4]	71.7	[66.6-76.2]	
**Scheduling of 1st Antenatal Check (in months)**					0.001
0-3 months	33.3	[29.0-37.8]	66.7	[62.2-71.0]	
4-6 months	49.3	[43.0-55.7]	50.7	[44.3-57.0]	
7-9 months	64.3	[40.6-82.6]	35.7	[17.4-59.4]	
**Perceived Susceptibility and Severity of Malaria**					0.897
Disagree	38.7	[33.4-44.2]	61.3	[55.8-66.6]	
Agree	39.1	[34.5-44.0]	60.9	[56.0-65.5]	
**Exposure to messages on IPTp-SP (in the past 6 months)**					0.052
No	39.9	[35.9-44.0]	60.1	[56.0-64.1]	
Yes	24.8	[14.4-39.4]	75.2	[60.6-85.6]	
***Community-Level***					
**Region**					0.016
Greater Accra	43	[34.5-51.9]	57	[48.1-65.5]	
Central	37.6	[29.5-46.3]	62.4	[53.7-70.5]	
Western	32.5	[25.4,40.4]	67.5	[59.6-74.6]	
Volta	46.8	[29.2-65.3]	53.2	[34.7-70.8]	
Eastern	57.8	[47.0-67.9]	42.2	[32.1-53.0]	
Ashanti	35.8	[29.0-43.2]	64.2	[56.8-71.0]	
Brong Ahafo	36.8	[26.2-48.9]	63.2	[51.1-73.8]	
Northern	35.5	[24.6-48.3]	64.5	[51.7-75.4]	
Upper East	22.6	[14.3-33.8]	77.4	[66.2-85.7]	
Upper West	22.2	[13.3-34.7]	77.8	[65.3-86.7]	
**Place of Residence**					
Urban	40.5	[35.5-45.8]	59.5	[54.2-64.5]	0.475
Rural	37.8	[32.5-43.4]	62.2	[56.6-67.5]	
**Community Exposure to Malaria Messages**	5.2	[3.3-7.1]	6.5	[4.8-8.3]	0.086
**Perceived Community Susceptibility and Severity of Malaria**	61.2	[56.3-66.2]	60.4	[55.9-64.9]	0.697
** Community Level of ANC Visits**	655.8	[623.5-688.0]	715.3	[690.0-740.8]	0.697
**Total**	39		61		

*p* values for all categorical variables were derived from the Pearson Chi-Squared tests, *p* values for all continuous variables (community exposure to malaria messages, perceived community susceptibility and severity of malaria, and community level of ANC visits) were derived from t-test.

Source: 2019 GMIS dataset

Generally, few explanatory variables were significantly associated with IPTp-SP, whereas others had no relationship with the result variable. A pregnant woman’s uptake of IPTp-SP was significantly linked with religion, region of residence, number of ANC visits, and scheduling of the first ANC visit. Approximately 45.1% of women who were Pentecostal/Charismatic Christians took less than three doses, while 74.4% of Catholics took three or more doses of IPTp-SP. More than half of women (57.8%) who resided in the Eastern Region took less than three doses, whereas most women (77.8%) of women in the Upper West took three or more doses of IPTp-SP. Regarding health-related characteristics, 71.9% of women who took less than three doses reported three or fewer ANC visits compared to 71.7% who took three or more doses of IPTp-SP and had eight or more ANC visits. Furthermore, scheduling of the first ANC visit showed that 64.3% of women whose first ANC visit was initiated within 7–9 months (third trimester) had less than three doses of IPTp-SP while 66.7% of them who took three or more doses of IPTp-SP were between 0-3 months (first trimester).

### Results of binary logistic regression analysis

In the binary logistic regression analysis, we used a total sample of 1,151 women between the ages of 15 and 49 with their most recent birth in the last two years before the survey to examine the magnitude of the relationship between individual and community level factors and IPTp-SP uptake. Table 3 shows the unadjusted and adjusted OR and CI of the explanatory variables of participants. In the unadjusted model, a pregnant woman’s uptake of IPTp-SP was significantly predicted by age, religion, region of residence, number of ANC visits, scheduling of first ANC visit, and community Level of ANC Visits. However, the region of residence, number of ANC visits, and scheduling of the first ANC visit significantly predicted IPTp-SP uptake in the adjusted model.

**Table 3:** Binary logistic regression of participants

Variables	Unadjusted		Adjusted	
	OR	CI	OR	CI
***Individual-Level: Socio-demographic***				
** Age (in years)**				
** 15-19**	Ref		Ref	
** 20-24**	1.1	0.6 - 2.0	1	0.5 - 1.7
** 25-29**	1.6	0.9 - 2.9	1.4	0.8 - 2.5
** 30-35**	1.6	1.0 - 2.6	1.6	0.9 - 2.6
** 35-39**	1.6	0.8 - 3.0	1.4	0.7 - 2.8
** 40+**	2.4*	1.0 - 5.5	1.9	0.8 - 4.4
**Educational Level**				
** None**	Ref		Ref	
** Primary**	0.6*	0.4 - 0.9	0.6	0.4 - 1.0
** Secondary and Higher**	1	0.6 - 1.5	1	0.6 - 1.8
**Ethnicity**				
** Akan**	Ref		Ref	
** Mole-Dagbani**	1.1	0.8 - 1.6	0.6	0.3 - 1.1
** Ewe**	0.8	0.5 - 1.3	1.3	0.8 - 2.2
** Other**	0.7	0.5 - 1.0	0.7	0.4 - 1.2
**Religion**				
** Catholic**	Ref		Ref	
** Pentecostal/Charismatic**	0.4**	0.2 - 0.7	0.5	0.3 - 1.0
** Other Christians**	0.6	0.4 - 1.1	0.8	0.4 - 1.6
** Muslim**	0.7	0.4 - 1.2	1	0.5 - 1.8
** Other**	0.5	0.2 - 1.4	0.7	0.3 - 1.9
** Wealth index **				
** Poorest**	Ref		Ref	
** Poorer**	1.1	0.7 - 1.6	1.2	0.7 - 2.0
** Middle**	1.1	0.7 - 1.7	1	0.6 - 1.8
** Richer**	1.2	0.7 - 1.9	1.3	0.7 - 2.6
** Richest**	1	0.6 - 1.7	0.9	0.4 - 1.9
***Individual-Level: Health-Related***				
** Number of Antenatal Visits During Pregnancy**				
** 0-3 visits**	Ref		Ref	
** 4-7 visits**	3.6***	2.0 - 6.5	3.3***	1.8 - 6.0
** 8+ visits**	6.5***	3.7 - 11.4	4.9***	2.6 - 9.3
**Scheduling of 1st Antenatal Check (in months)**				
** 0-3 months**	Ref		Ref	
** 4-6 months**	0.5***	0.4 - 0.7	0.7*	0.5 - 1.0
** 7-9 months**	0.3*	0.1 - 0.8	0.9	0.3 - 2.6
**Perceived Susceptibility and Severity of Malaria**				
** Disagree**	Ref		Ref	
** Agree**	1	0.7 - 1.3	1.1	0.8 - 1.6
**Exposure to messages on IPTp-SP (in the past 6 months)**				
** No**	Ref		Ref	
** Yes**	2	1.0 - 4.1	1.6	0.6 - 4.0
***Community-Level***				
** Region**				
** Greater Accra**	Ref		Ref	
** Central**	1.3	0.8 - 2.1	1.4	0.7 - 2.9
** Western**	1.6	1.0 - 2.6	1.3	0.7 - 2.5
** Volta**	0.9	0.4 - 2.0	1.2	0.5 - 2.9
** Eastern**	0.6*	0.3 - 1.0	0.7	0.3 - 1.4
** Ashanti**	1.4	0.8 - 2.2	1.5	0.8 - 2.7
** Brong Ahafo**	1.3	0.7 - 2.4	1.7	0.9 - 3.1
** Northen**	1.4	0.7 - 2.6	1.9	0.9 - 4.1
** Upper East**	2.6**	1.3 - 5.0	3.0*	1.2 - 7.3
** Upper West**	2.6**	1.3 - 5.4	5.3**	1.9 - 14.7
**Place of Residence**				
** Urban**	Ref		Ref	
** Rural**	1.1	0.8 - 1.5	1.2	0.8 - 1.8
** Community Exposure to Malaria Messages**	3.3	0.7 - 16.7	1	0.1 - 9.8
** Perceived Community Susceptibility and Severity of Malaria**	0.9	0.5 - 1.7	1.4	0.6 - 3.1
** Community Level of ANC Visits**	1.3***	1.1 - 1.4	1.2	1.0 - 1.4
**Total**	61		39	

***p<0.001, **p<0.01, *p<0.05

Source: 2019 GMIS dataset

Among the individual-level socio-demographic factors in the unadjusted model, women aged 40 and older had 2.4 times higher odds of IPTp-SP Uptake than those between the ages of 15 and 19. Pentecostal/Charismatic women had 60% lower odds of IPTp-SP Uptake than Catholics. The unadjusted individual-level health-related model showed that women with 4–7 ANC visits during pregnancy had 3.6 times higher odds of IPTp-SP Uptake than those with 0–3 visits. A similar trend was reported in the adjusted model, where women with 4–7 ANC visits had 3.3 times higher odds of IPTp-SP Uptake than those with 0–3 visits. Likewise, participants with eight or more ANC visits had 4.9 times higher odds of IPTp-SP Uptake than women with 0–3 visits. In the unadjusted model, women who attended their first ANC in the second and third trimesters had 50% and 70% lower odds of IPTp-SP Uptake, respectively, than those in their first trimesters. Besides, the adjusted model showed that only women in the second trimester had 30% lower odds of IPTp-SP Uptake. Aside from individual factors, pregnant women in the Eastern Region had 40% lower odds than those in the Greater Accra Region. Nonetheless, participants in the Upper East and Upper West Regions had 2.6 times each higher odds of IPTp-SP Uptake than those in the Greater Accra Region. In the adjusted individual-level socio-demographic model, only residents in the Upper East and Upper West Regions had 3.0 and 5.3 times higher odds of IPTp-SP Uptake than those in the Greater Accra Region, respectively. Additionally, the community level of ANC visits during pregnancy had 1.3 times higher odds of IPTp-SP Uptake in the unadjusted model.

## Discussion

Our study examined the individual and community-level predictors of IPTp-SP uptake among pregnant women in Ghana using the 2019 GMIS. The study’s findings showed that 61% of pregnant women in Ghana received optimal (3+) doses of IPTP-SP, while 39% received none/partial (< 3) doses of IPTp-SP. This 61% optimal IPTp-SP uptake by the women was higher compared to the prevalence reported in other Sub-Saharan African countries like 13% in Malawi [31], 16.8% in Nigeria [32], 25% in Uganda [31], 31% in Sierra Leone [31], 37% in Kenya [31], 43% in Tanzania [33], and 60% in Zimbabwe [28]. Nonetheless, our study’s prevalence estimate of optimal uptake (61%) in Ghana is slightly lower than the country’s 2016 IPTp-SP optimal uptake estimate of 63% using the GMIS by Darteh et al. [10]. Furthermore, the prevalence of 61% of optimal doses in this present study is lower than the target of the National Malaria Control Programme (NMCP) of to reduce the malaria morbidity and mortality burden by 75% (baseline 2012) by the year 2020 and the global target of 80% for IPTp-SP uptake of at least two or more doses [34, 35]. 

Our results also indicated that IPTP-SP uptake tends to be associated with the religion of pregnant women. This study supports evidence from previous research on the relationship between religious beliefs and IPTp-SP uptake in Ghana among pregnant women [10]. The importance of religion in IPTp-SP uptake in Africa cannot be underestimated, as it drives socialization and health behaviors [10, 36]. 

The role of ANC attendance could be viewed from two perspectives in the present study. We observed pregnant women’s schedule of first ANC visits and the number of ANC visits as vital to IPTp-SP uptake. Concerning the timing (month) of ANC visit, pregnant women who initiated their first ANC visit between the first three months of gestation were more likely to take optimal IPTp-SP dose than those who had their first visit between the second and third trimesters months of pregnancy. For example, several studies have also shown that the timing of the first ANC visit is associated with IPT uptake [21, 26, 28, 36–39]. Thus, pregnant women who first attended ANC when they were between 4 and 6 months were less likely to take the optimal doses of SP compared to those who visited during the first trimester [26]. 

Also, our findings aligned with studies indicating that the number of ANC visits strongly predicts IPTp-SP uptake [39, 40]. The findings showed that pregnant women with four or more ANC visits had more chances to take optimal IPTp-SP doses than those who reported fewer ANC visits. Since IPTp-SP medication is obtained during routine ANC visits, pregnant women with four or more visits may have more chances to meet the minimum three doses required for optimal uptake. Besides, Buh et al. [39] and Martin et al. [40] also reported that ANC visits were strongly associated with IPT uptake. Since IPTp-SP was typically provided during ANC visits, the frequency of attendance matches women’s chances to receive chemoprophylaxis. Moreover, ANC attendance allowed healthcare providers to administer IPTp-SP and offer education to reinforce the importance of adherence to the regimen [10, 21, 41]. 

The study found no significant associations between covariates of pregnant women like age, educational level, ethnicity, wealth quintile and IPTp-SP uptake. The findings related to educational level and wealth quintile, in particular, were unanticipated given that past studies conducted in SSA countries, including Sierra Leone, Democratic Republic of Congo, Madagascar, Mozambique and Nigeria and have reported that socioeconomic factors such as wealth status and education may also play a role in optimal IPTp-SP dose [39, 42]. Another interesting finding was that perceived susceptibility and severity of malaria had no significant association with IPTp-SP uptake in the current study. This finding agrees with results obtained by Awantang et al. [43], indicating that perceived severity and susceptibility to malaria are not associated with higher odds of optimal uptake of IPTP-SP among pregnant women. Possible explanations could be that some pregnant women in this context lack adequate knowledge of the IPTp-SP regimen. Thus, they do not perceive themselves to be at high risk of contracting malaria nor perceived the disease as severe. Again, some women may perceive IPTp-SP as harmful or unnecessary.

Regarding community-level characteristics, a significant association between IPTP-SP uptake and the region of residence of pregnant women. Compared to the Greater Accra Region, the regression analysis showed residents in the Upper East and Upper West Regions were more likely to have optimal uptake. These findings support the findings of Darteh et al. [10] and Doku et al. [21], who indicated that pregnant women from Northern Ghana are more likely to receive optimal doses of IPTp-SP compared to other regions. This finding may be due to the high intensity of malaria preventive interventions often targeted at the northern communities [37]. 

Additionally, findings in our study indicated that the place of residence of pregnant women had no significant association with IPTp-SP uptake. This result seems consistent with other research that reported that the association between place of residence and intermittent preventive treatment in pregnancy with IPTp-SP uptake might vary depending on the context and specific factors [10, 28]. Therefore, it is essential to consider context-specific factors such as region of residence when examining the relationship between place of residence and IPTp-SP uptake. Aside from this relationship, our study found no significant IPTp-SP uptake differences among the other community-level factors like exposure to malaria messages, perceived susceptibility and severity, and level of ANC visits.

These findings contradicted those of previous studies that stated that exposure to malaria messages and community norms and beliefs towards IPTp-SP could influence IPTp-SP uptake [10, 21, 44]. This somewhat contradictory result may be due to findings like the timing and frequency of similar ANC visits, which might have dominated the different communities. Also, using a single item to explore behavioural health indicators may not measure the attribute adequately. Previous research has reported significant associations between community exposures to malaria messages, community perceived susceptibility, the severity of malaria and place of residence in the uptake of IPTp-SP in pregnancy [10, 21, 44]. This inconsistency probably showed the potency of individual-level factors influencing optimal IPTp-SP uptake rather than community-related variables. While these community-level factors may not predict optimal IPTp-SP dose, their essence in promoting optimal IPTp-SP uptake among pregnant women cannot be overlooked.

## Conclusion

Ghana has slightly declined in the gains made in 2016 regarding the optimal uptake of IPTp-SP among pregnant women. Our study found that the region of residence of pregnant women predicts IPTP-SP uptake. Aside from women in the Upper East and Upper West Regions who reported increased chances of IPTp-SP Uptake than those in the Greater Accra Region, which is the capital of Ghana, all other regions need interventions to strengthen IPTp-SP Uptake than those in the Greater Accra Region, all other regions need interventions to services. Additionally, pregnant women who initiated their ANC in the first trimester and attended ANC 4 or more times indicated the value of early initiation and frequency of ANC attendance in the IPTP-SP uptake program.

We recommend that the Ministry of Health, Ghana Health Service, the National Malaria Control Programme, and other malaria prevention and control stakeholders consider these factors to improve service delivery and uptake of IPTP-SP services. Strategies and policies to enhance the uptake of optimal doses of IPTp-SP in Ghana should include engagement of religious leaders in IPTp-SP campaigns, tailoring information and messages on IPTp-SP to fit early initiation of first ANC and increasing ANC visits during pregnancy. Also, interventions and policies at all levels of healthcare should promote positive perceptions of ANC services at the regional level. Furthermore, equitable access and budgetary allocations to cover ANC services to cover additional interventions in regions of high levels of none/partial uptake.

## Limitation

Aside from the valuable contributions this study makes to the evidence on IPTp-SP uptake, the following limitations must be considered when interpreting the data. First, the study used secondary data, which does not give researchers the flexibility to measure variables of interest. Second, using a single item to measure variables like individual perceived susceptibility to malaria infection may not fully capture the complete picture of constructs. Third, the categorization of variables, for example, the category “4–7 visits as a predictor of IPTp-SP uptake,” does not imply that four visits are ideal. From a health systems perspective, women attending at least four visits may still not receive the optimal care needed to ensure the best maternal and child health outcomes. Nonetheless, the MIS is among the best surveys showing a national IPTp-SP uptake across time and space.

## Data Availability

All data generated or analyzed during this study are available from the DHS Program’s website (https://dhsprogram.com/data/dataset_admin/index.cfm) upon request.

## Abbreviations

DHS: Demographic and Health Survey
GMIS: Ghana Malaria Indicator Survey
ITN: Insecticide-treated bed nets
SBCC: Social and behavior change communication
IPTp-SP: Intermittent preventive treatment with sulfadoxine-pyrimethamine
SSA: Sub-Saharan Africa
SDG: Sustainable Development Goal
ANC: Antenatal Care
WHO: World Health Organization
